# Broadening the biocompatibility of gold nanorods from rat to *Macaca fascicularis*: advancing clinical potential

**DOI:** 10.1186/s12951-021-00941-1

**Published:** 2021-06-30

**Authors:** Jinfeng Liao, Taorang Tian, Sirong Shi, Xueping Xie, Shuanglin Peng, Ying Zhu, Jingang Xiao, Yunfeng Lin

**Affiliations:** 1 State Key Laboratory of Oral Diseases, National Clinical Research Centre for Oral Diseases, West China Hospital of Stomatology, Sichuan University, Chengdu, 610041 China; 2 Department of Oral and Maxillofacial Surgery, The Affiliated Stomatology Hospital of Southwest Medical University, Luzhou, 646000 China; 3 Division of Physical Biology, CAS Key Laboratory of Interfacial Physics and Technology, Shanghai Synchrotron Radiation Facility, Shanghai Institute of Applied Physics, Chinese Academy of Sciences, Shanghai, 201800 China; 4 Zhangjiang Laboratory, Shanghai Advanced Research Institute, Chinese Academy of Sciences, Shanghai, 201210 China; 5 College of Biomedical Engineering, Sichuan University, Chengdu, 610041 China

**Keywords:** Gold nanorods, Systemic biocompatibility, *Macaca fascicularis*, Blood clearance, Biodistribution, Major organ analysis

## Abstract

**Background:**

The biomedical field has used gold nanorods (GNRs) for decades; however, clinical trials and translation is limited except gold nanoshells. The preparation of gold nanoshells is more complex than that of polyethylene glycol-modified GNRs (PEG-GNRs), and it is difficult to ensure uniform thickness. It is important to encourage and broaden the use of the star member (PEG-GNRs) of gold nanoparticles family for clinical translation. Existing studies on PEG-GNRs are limited with no relevant systematic progression in non-human primates. Herein, we assessed the systematic biocompatibility of PEG-GNRs in rats and clinically relevant *Macaca fascicularis*.

**Results:**

In this small animal study, we administrated multiple doses of PEG-GNRs to rats and observed good biocompatibility. In the non-human primate study, PEG-GNRs had a longer blood half-life and produced a negligible immune response. Histological analysis revealed no significant abnormality.

**Conclusions:**

PEG-GNRs were well-tolerated with good biocompatibility in both small animals and large non-human primates. The information gained from the comprehensive systemic toxicity assessment of PEG-GNRs in *M. fascicularis* will be helpful for translation to clinical trials.

**Graphical abstract:**

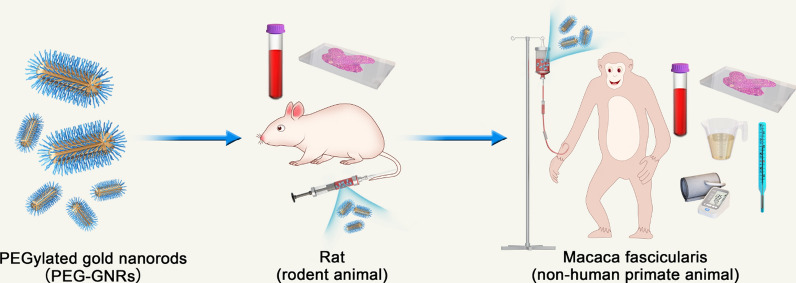

**Supplementary Information:**

The online version contains supplementary material available at 10.1186/s12951-021-00941-1.

## Introduction

Gold nanoparticles are undergoing rapid development, and their unique properties give them great potential for clinical applications such as drug carriers, diagnostic tools, and therapeutic agents. A clinical pilot study employed gold nanoshells in a photothermal therapy approach to treat prostate tumors [1]. Gold nanoparticles are promising in clinical and biomedical applications, such as gold nanorods (GNRs) as nanocarriers and photothermal agents. Murphy’s group first invented and synthesized GNRs [2, 3]. GNRs can be prepared via a well-established, reproducible seed-mediated route. GNRs are simple to produce, and they possess long-time colloidal stability. However, the residual cetyltrimethylammonium bromide (CTAB) used as a template during GNRs synthesis is highly toxic [4]. The residual CTAB needs to be replaced to improve the biocompatibility of GNRs [5, 6]. Polyethylene glycol (PEG) is a classic candidate to replace CTAB for GNRs modification [7, 8] and PEG have been used in clinical translation under the approval the Food and Drug Administration. PEG modification is a potential strategy for improving stability, reducing toxicity, and enhancing membrane transport [9, 10]. GNRs are rod-shaped, with unique longitudinal and transverse surface plasmon resonance [11, 12] as well as an adjustable aspect ratio which allows near-infrared region (NIR) absorption [13, 14]. These properties confer two unique advantages to PEG-modified GNRs (PEG-GNRs): 1) their synthesis is easier and more controllable than that of gold nanoshell and 2) their NIR adsorption is adjustable by their aspect ratios. PEG-GNRs have multiple scientific applications [15] such as photothermal therapy agents, [16–20] bioimaging agents, [21–24] drug carriers, [25–27] gene delivery agents, [28, 29] and biosensors [30–32]. These continued developments give great potential for their clinical use. Unfortunately, PEG-GNRs research has remained theoretical, and no preclinical studies in large animals exist. Different shapes of PEG-GNRs and gold nanoshells may produce different responses in the body. Thus, studies on the matter of PEG-GNRs are required.

Previous in vivo toxicity studies of PEG-GNRs have focused on rodents, because numerous nanomedicines have been used successfully in small animals but failed in clinical practice, [31] and the toxicity results from small animals alone are not reliable. The systemic toxicity of some heavy metal and organic nanoparticles such as CdSe/CdS/ZnS quantum dots, [34, 35] silica quantum dots, [36] detonation nanodiamonds, [37] and graphene oxide [38] have been studied in large animals. In one recent study, anaphylactic death of non-human primates occurred with graphene oxide but not with single-walled carbon nanotubes and nanodiamonds [39]. These studies highlight the great importance of assessing nanotoxicity in large animals. A broadening systemic understanding of biocompatibility in non-primate animals is desperately needed to bring GNRs into clinical research.

In this study, we systemically assessed the toxicity of PEG-GNRs in rat and *Macaca fascicularis*, because of their genetic similarity to humans. We treated *M. fascicularis* with PEG-GNRs and assessed their blood pharmacokinetic parameters, biodistribution, blood immune response, urinalysis, and histology. We observed no significant changes between the PEG-GNRs-treated group and control group, except a negligible immune response that was well-tolerated. Histological analysis revealed no severe toxicity. The remaining inorganic nanomaterial in the reticuloendothelial system (RES) showed low short-term breakdown and clearance of PEG-GNRs. The biocompatibility assessment in higher mammals furthers our understanding of the biosafety of PEG-GNRs, which may support the development of their clinical application.

## Results and discussion

**Fig. 1:**
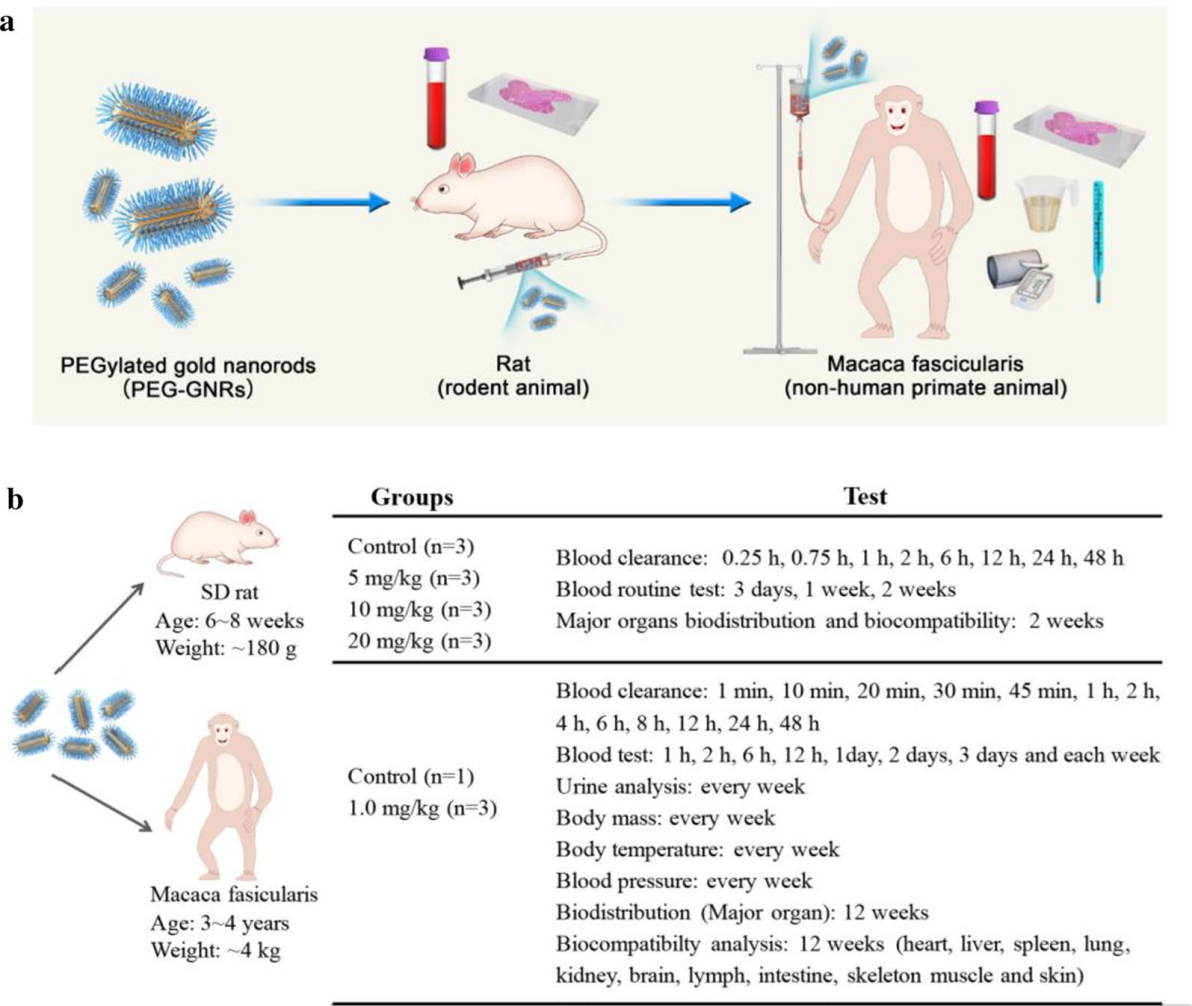
**a** The systemic biocompatibility study on PEG-GNRs in rats and *Macaca fasicularis*. **b** Experiment design

There are four steps in the research and development of new nanomedicine agents: discovery, preclinical research, clinical research, and new drug declaration. The role of preclinical research is to ensure the non-toxicity of compounds before starting human trials [37]. For further development of gold nanorods, we assessed their systemic biocompatibility in rodents and non-primate animals. Figure 1a shows how the prepared PEG-GNRs were injected in rats and *M. fascicularis* and biocompatibility was assessed in blood, tissue, and urine samples. Our detailed experimental design is shown in Fig. 1b. Studies in the Spraque–Dawley (SD) rats included pharmacokinetic clearance, standard blood measures, and major organ biocompatibility. The studies in *M. fascicularis* included blood measures, urinalysis, and measures of body mass, body temperature, blood pressure, and tissue distributions at designated time points.

**Fig. 2:**
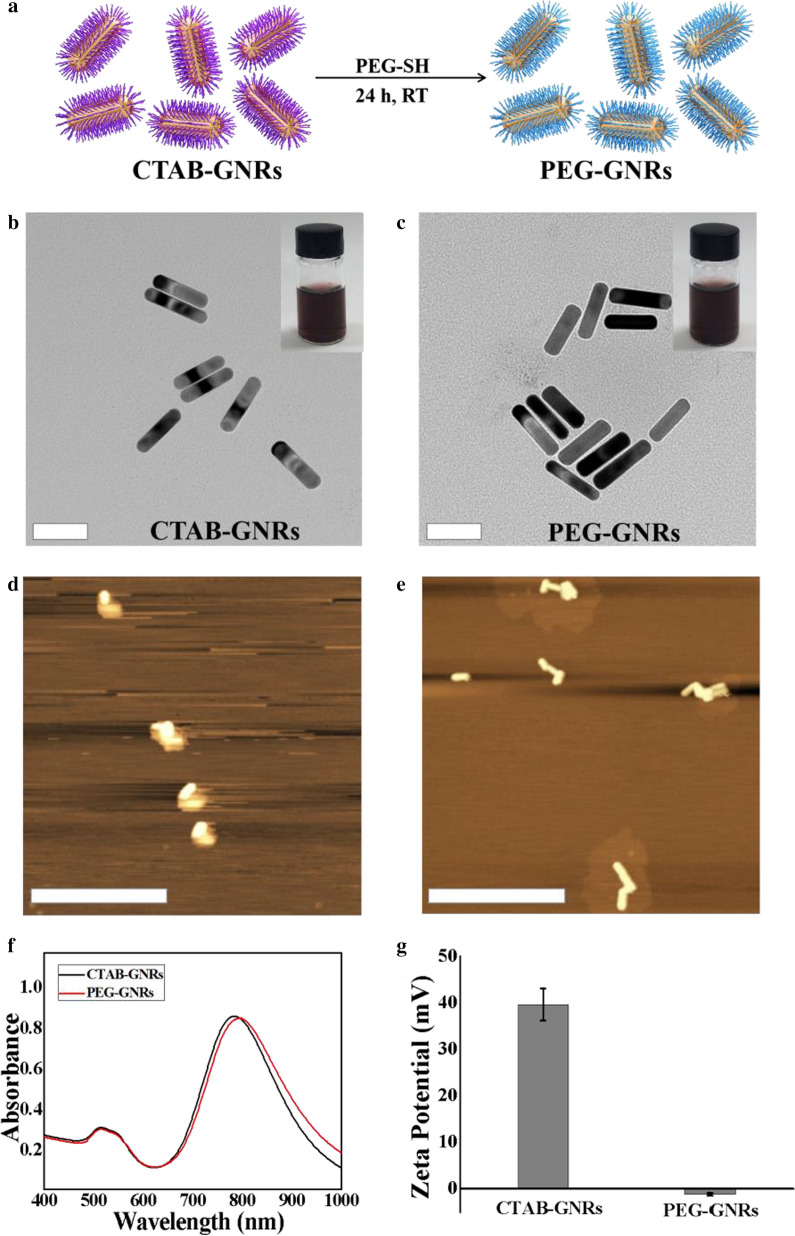
**a** The PEG-GNRs synthesis scheme. TEM images of (**b**) CTAB-GNRs and **c** PEG-GNRs. (Scale bars = 50 nm. The inset pictures are the photographs of CTAB-GNRs and PEG-GNRs.) AFM images of (**d**) CTAB-GNRs and **e** PEG-GNRs. (Scale bars = 500 nm) (**f**) UV–Vis absorbance spectra of CTAB-GNRs and PEG-GNRs. **g** *Zeta* potentials of CTAB-GNRs and PEG-GNRs

We prepared the PEG-GNRs in two steps. After synthesizing the CTAB-GNRs using a modified seed-mediated approach, we PEGylated them with PEG-SH (Fig. 2a). The prepared CTAB-GNRs possess an aspect ratio of approximately 3.9 (length: ~ 47 nm, width: ~ 12 nm), as shown in the transmission electron microscopy (TEM) image (Fig. 2b). Replacing CTAB with PEG-SH via Au–S bonds formation yielded the PEG-GNRs. In Fig. 2c, the PEG coating is visible as a faint halo around the GNRs, which is not present on CTAB-GNRs. The color of their dispersions was not different (inset photographs in Fig. 2b, c). The atomic force microscopy (AFM) images also demonstrate the morphology of CTAB-GNRs (Fig. 2d) and PEG-GNRs (Fig. 2e). The maximum absorption wavelengths of the CTAB-GNRs and PEG-GNRs were in the NIR region, at approximately 800 nm and 805 nm, respectively (Fig. 2f). The *zeta* potential of PEG-GNRs was − 1.2 mV, whereas that of CTAB-GNRs was at + 39.6 mV (Fig. 2g). The replacement of CTAB with PEG greatly altered the surface charge.

**Fig. 3:**
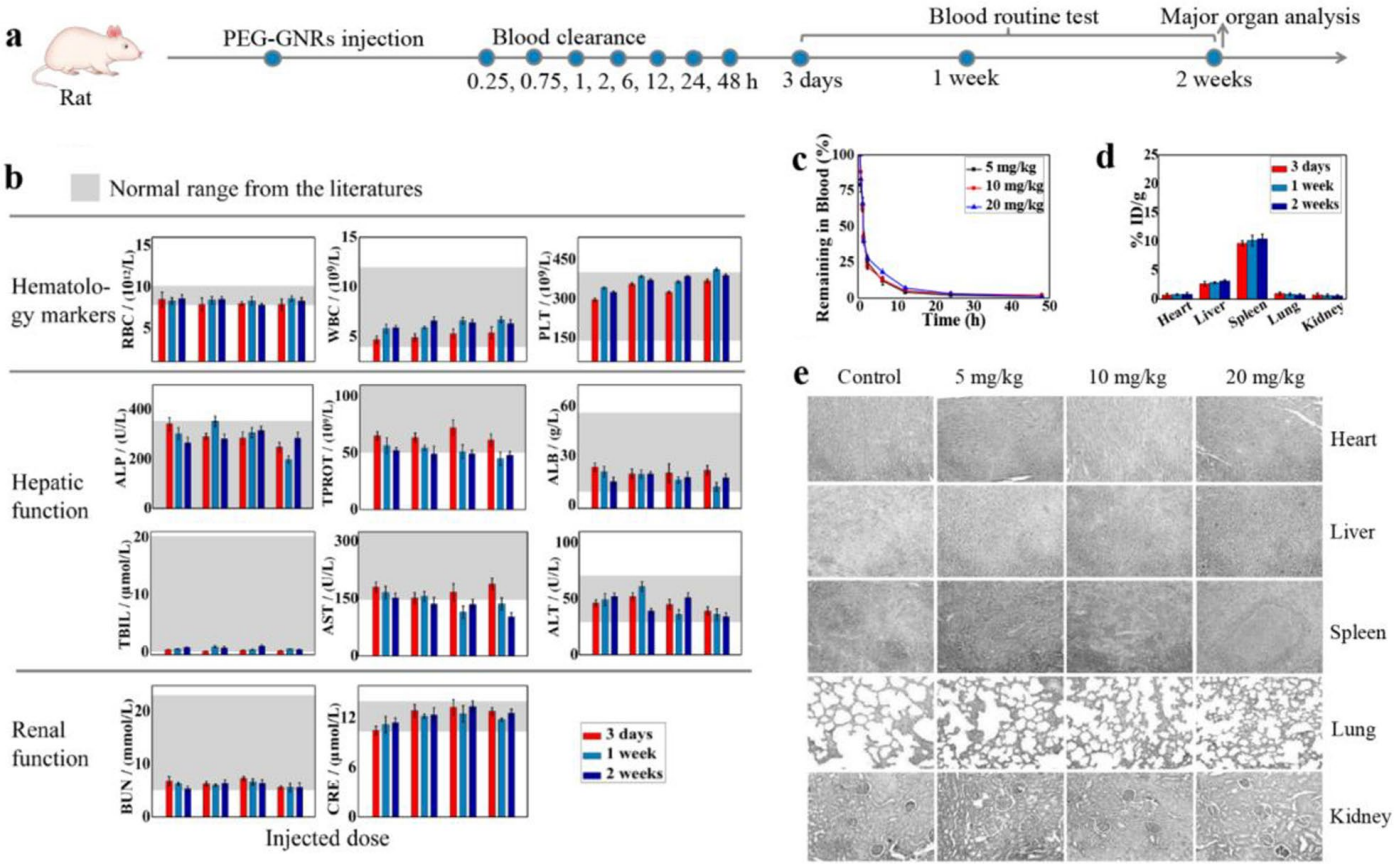
**a** Timeline of the PEG-GNRs injection and toxicity evaluations in rats. **b** The blood markers test of mice treated with PEG-GNRs (injected doses from left to right: 0 mg/kg, 5 mg/kg, 10 mg/kg, 20 mg/kg) at 3 days, 1 week, 2 weeks after treatment. In each histogram, the light gray regions represent the normal range according to the literature. *RBC *red blood cell,* WBC* white blood cell,* PLT* platelet,* ALP* alkaline phosphatase,* TPROT* total protein,* ALB* albumin,* TBIL* total bilirubin,* AST* aspartate transaminase,* ALT* alanine transaminase,* BUN* blood urea nitrogen,* CRE* creatinine. **c** The remaining Au in blood after PEG-GNRs injection. **d** The biodistribution at 3 days, 1 week, 2 weeks after systemic administration of 10 mg/kg PEG-GNRs in rats. **e** H&E staining photographs of major organs in rats 2 weeks after the treatment. Images were acquired at 200 × magnification

Next, we assessed the systemic toxicity of PEG-GNRs in rats and monkeys. Figure 3a shows the blood clearance, tissue distribution, and organ toxicity of PEG-GNRs in rats. As shown in Fig. 3b, blood markers and hepatic/renal function indicators were within normal range, with no substantial differences between the treated and control groups.

To quantitatively assess the blood circulation time and biodistribution of PEG-GNRs, we measured the remaining amount of gold (Au) in blood samples and tissues via inductively coupled plasma-atomic emission spectroscopy (ICP-AES). PEG-GNRs cleared from the blood with a longer half-life (t_1/2_ =  ~ 53 min) (Fig. 3c) than that of CTAB-GNRs (t_1/2_ < 15 min) cleared from the blood [40–42]. After 24 h, there was no residual Au in the blood. We evaluated the biodistribution of PEG-GNRs in rats at 3 days, 1 week, and 2 weeks after the treatment. Our results show that PEG-GNRs were mainly localized in the spleen and liver (Fig. 3d and Additional file 1: Figure S1). Although some doubts remain regarding the distribution of gold nanorods in the RES, [43–45] because the spleen is the major part of the RES, it can sequester foreign compounds entering the body. The concentration of Au in the spleen was almost 3.5-fold (average of the three test groups) higher than in the liver, and the relative concentrations of Au in the spleen and liver decreased with increasing doses. The ratios of Au in the spleen and liver were approximately 3.9, 3.5, and 3.0 for a dose of 5, 10, and 20 mg/kg, respectively. We hypothesize that when the spleen reaches saturation, the remaining Au is selectively distributed to the liver.

Although PEG-GNRs are mainly distributed in the RES histological analysis revealed no apparent structural change or abnormality in the spleen and liver (Fig. 3e and Additional file 1: Figure S2). Examination of the heart, lungs, and kidneys also showed no structural alteration and confirmed the absence of toxicity in vivo. No mortality and adverse effects on behavior occurred in the animals, even when treated with a maximal dose of 20 mg/kg PEG-GNRs.

**Fig. 4:**
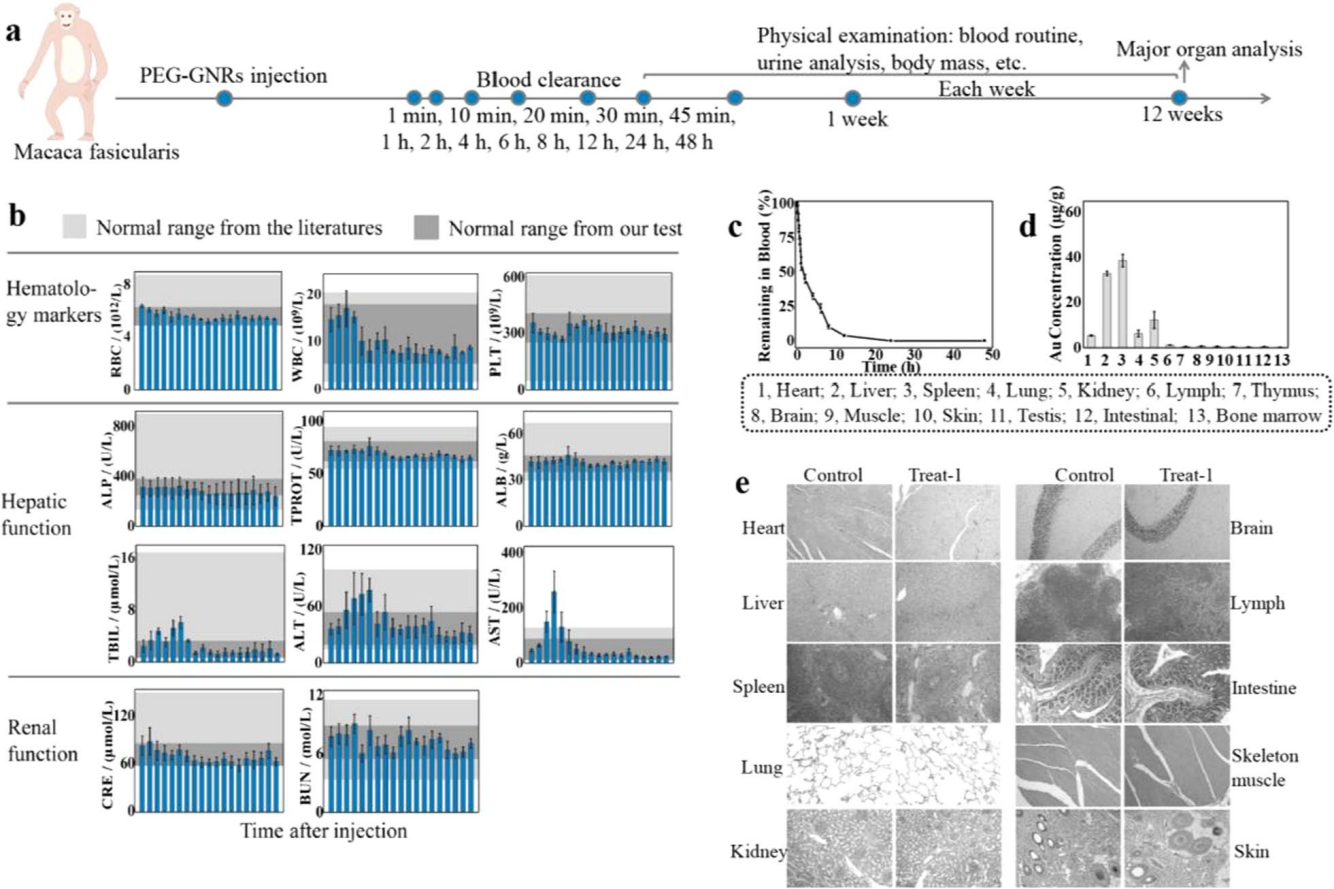
**a** Timeline of the PEG-GNRs injection and toxicity evaluations in Macaca fascicularis. **b** Blood test results. Times after injection in the figures from left to right are 1 h, 2 h, 6 h, 12 h, 1 d, 2 d, 3 d, 1 w, 2 w, 3 w, 4 w, 5 w, 6 w, 7 w, 8 w, 9 w, 10 w, 11 w, and 12 w. The light gray boxes indicate literature reference values, and the gray boxes indicate the control group in our test. **c** Blood circulation time of PEG-GNRs analyzed by measuring the remaining Au content in blood. **d** The biodistribution of PEG-GNRs in different organs at 12 weeks after treatment. **e** Histological images of the major organs of the control and experimental animal #1 (number 1) 12 weeks post-injection with PEG-GNRs. Images were acquired at 200 × magnification

Next, we conducted a pilot study of PEG-GNRs in *M. fascicularis* (Fig. 4a). The experimental group (n = 3) was administered PEG-GNRs (1.0 mg/kg) by intravenous drip over 30 min. Peripheral blood samples were collected at designated time points. As shown in Fig. 4b, the white blood cell (WBC) count increased during the first 12 h but did not exceed the normal range. Within 2 days after injection, alanine aminotransferase (ALT) and aspartate aminotransferase (AST) levels increased. Then, at 3 days, levels were restored to the normal range compared with the control group (gray boxes) and literature reference values (light gray boxes). WBCs increased within 1 day, and then gradually returned to normal levels. These results suggest that PEG-GNRs induced a slight immune response in the early stage after treatment. Because GNRs are most likely identified as foreign elements, an increase in WBC was expected. ALT increased but stayed within the normal range for 2 days. AST increased beyond the normal range, but the difference was not significant. Slight changes in these markers suggest that the liver acts as an important detoxification organ to tolerate PEG-GNRs [46]. The other kidney function and blood markers (such as neutrophils, lymphocytes, monocytes, eosinophils, and basophils: Additional file 1: Figure S3) were within normal ranges. Although a minor immune response occurred early after injection, we observed no apparent signs of allergic or toxic reactions.

To determine the blood clearance of PEG-GNRs, we collected blood samples at designated time points. Figure 4c shows the ratio of remaining Au in the blood clearance profile. The Au blood half-life was approximately 84 min. At 12 h post-injection, the remaining amount of Au in the blood reached 3.89%. The blood half-life of PEG-GNRs in *M. fascicularis* was longer than that in the rat. We also determined the remaining concentrations of Au in the major organs 12 weeks after injection and found PEG-GNRs accumulated predominantly in the spleen and liver (Fig. 4d), similar to the results in the rodent. Next, we collected the major organs and tissues such as heart, liver, spleen, lung, kidney, brain, lymph, intestine, skeleton muscle, and skin for hematoxylin and eosin (H&E) histological staining sections (Fig. 4e and Additional file 1: Figures S4, Figure S5). We found no significant changes in these organs, even in the liver and spleen, where the PEG-GNRs accumulated and thus observed no signs of PEG-GNRs-induced tissue damage. According to previous studies, [47–49] the main route of nanoparticles clearance is through the extravascular extracellular space (EES), and the small particles (< 9 nm) are eliminated through the kidney. Based on biodistribution results, PEG-GNRs clearance might occur gradually through the EES [50, 51]. Although PEG-GNRs showed no toxicity in rats and *M. fascicularis*, long-term (1 or 2 year) studies are needed to determine their ultimate elimination route and satisfy clinical safety concerns [52, 53].

**Fig. 5:**
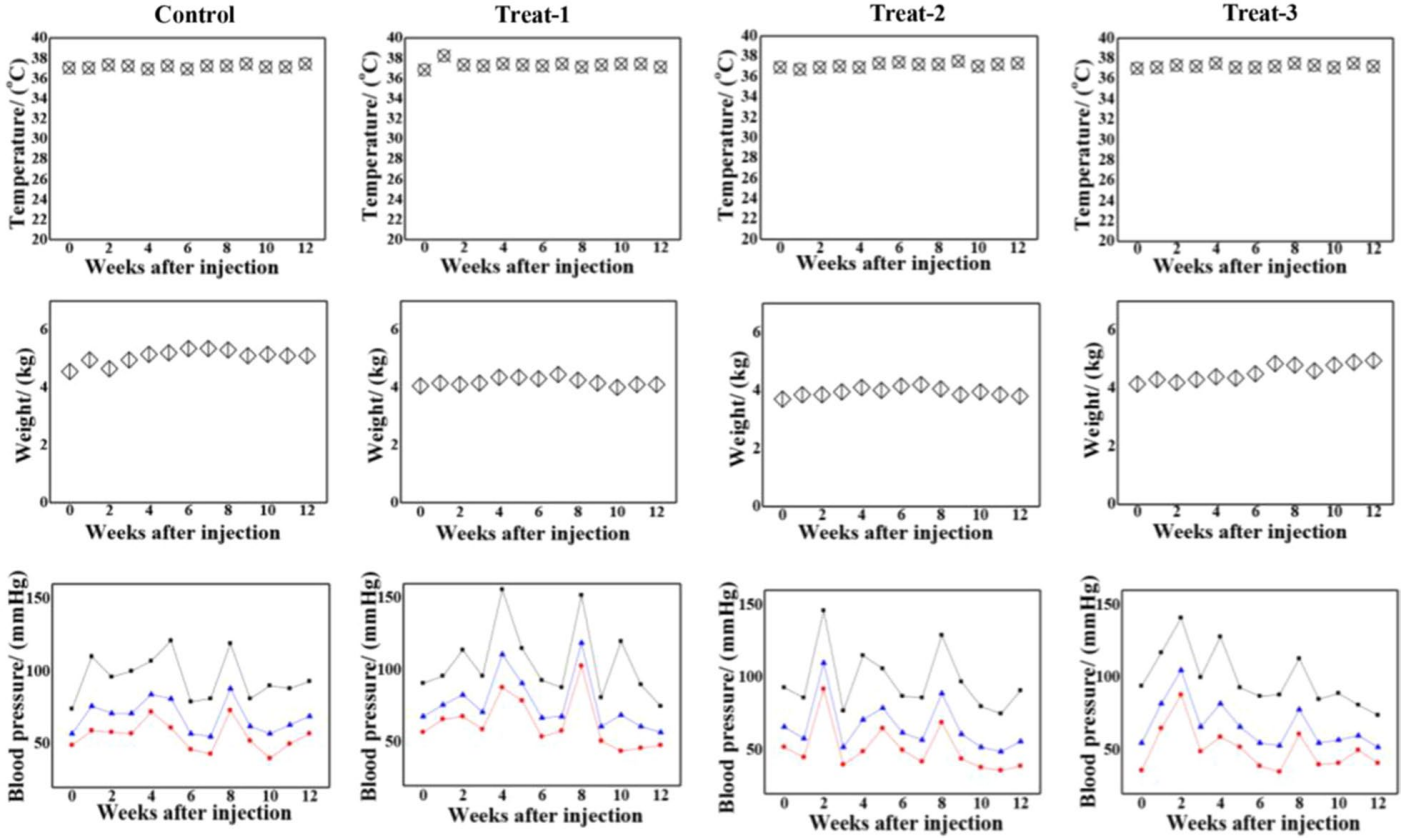
Body temperature, body weight, and blood pressure of control and the three treated *Macaca fascicularis* throughout the study. In the blood pressure curves, the black, blue, and red lines indicate systolic pressure, mean pressure, and diastolic pressure, respectively [Mean pressure = 1/3 (systolic pressure + 2 diastolic pressure)]

Next, we performed urinalysis in control (Table 1) and experimental animals (Tables 2, 3, and 4). All urine markers were within the normal range and the urine samples were yellow and clear, suggesting functional bladder and kidneys. We monitored other body signs such as body temperature, body weight, and blood pressure weekly (Fig. 5). There were no instances of fever or abnormal temperature. Body weight increased gradually over time, and blood pressure remained almost within the normal range. The animals’ activities were supervised and recorded throughout the 12-week evaluation period and showed no abnormal behaviors or vomiting. Thus, the results indicate that PEG-GNRs were relatively safe and well-tolerated in a non-human primate animal.

**Table 1:** Urine test of the control *Macaca fascicularis* in 12 weeks

Date week	GLU mmol/L	BIL μmol/L	KET mol/L	SG	BLO Ery/μL	pH	PRO g/L	URO μmol/L	NIT	LEU leu/μL	Color	Clarity
0	–	–	3.9	1.01	–	7.5	–	3.2	+	–	Yellow	Clear
1	–	–	–	1.02	–	8.5	–	3.2	–	–	Yellow	Clear
2	–	–	–	1.02	Trace	≥ 9	–	3.2	–	–	Yellow	Clear
3	–	–	–	1.02	Trace	≥ 9	–	3.2	–	–	Yellow	Clear
4	–	–	–	1.02	–	8.5	0.3	3.2	–	–	Yellow	Clear
5	–	–	–	1.02	–	≥ 9	–	3.2	–	–	Yellow	Clear
6	–	–	–	1.02	–	8.5	–	3.2	–	–	Yellow	Clear
7	–	–	–	1.02	–	≥ 9	–	3.2	–	–	Yellow	Clear
8	–	–	–	1.02	–	≥ 9	–	3.2	–	–	Yellow	Clear
9	–	–	–	1.02	–	≥ 9	0.3	3.2	+	–	Yellow	Clear
10	–	–	–	1.01	–	8.5	0.3	3.2	+	–	Yellow	Clear
11	–	–	–	1.01	–	≥ 9	1	3.2	–	–	Yellow	Clear
12	–	–	–	1.02	–	8.0	1	3.2	–	–	Yellow	Clear

*GLU* Glucose, *BIL* Bilirubin, *KET* Ketone, *SG* Specific Gravity, *BLO* Occult Blood, *PRO* Protein, *URO* Urobilinogen, *NIT* Nitrite, *LEO* Leukocyte

**Table 2:** Urine test of the PEG-GNRs treated *Macaca fascicularis* (number 1) in 12 weeks

Date Week	GLU mmol/L	BIL μmol/L	KET mol/L	SG	BLO Ery/μL	pH	PRO g/L	URO μmol/L	NIT	LEU leu/μL	Color	Clarity
0	–	–	3.9	1.01	5	≥ 9	–	3.2	–	–	Yellow	Clear
1	–	–	–	1.02	–	8.5	–	3.2	–	–	Yellow	Clear
2	–	-	–	1.02	Trace	≥ 9	–	3.2	-	–	Yellow	Clear
3	–	–	–	1.02	Trace	8.5	–	3.2	+	–	Yellow	Clear
4	–	–	–	1.02	–	≥ 9	–	3.2	-	–	Yellow	Clear
5	–	–	–	1.02	–	≥ 9	–	3.2	–	–	Yellow	Clear
6	–	–	–	1.02	Trace	≥ 9	Trace	3.2	Trace	–	Yellow	Clear
7	–	–	-	1.02	–	≥ 9	–	3.2	–	–	Yellow	Clear
8	–	–	–	1.02	–	≥ 9	–	3.2	–	–	Yellow	Clear
9	–	–	Trace	1.02	–	8.5	–	3.2	–	–	Yellow	Clear
10	–	–	–	1.02	–	8.5	–	3.2	–	–	Yellow	Clear
11	–	–	-	1.01	–	8.5	–	3.2	–	–	Yellow	Clear
12	–	–	-	1.02	–	8.5	–	3.2	–	–	Yellow	Clear

*GLU* Glucose, *BIL* Bilirubin, *KET* Ketone, *SG* Specific Gravity, *BLO* Occult Blood, *PRO* Protein, *URO* Urobilinogen, *NIT* Nitrite, *LEO* Leukocyte

**Table 3:** Urine test of the PEG-GNRs treated *Macaca fascicularis* (number 2) in 12 weeks

Date Week	GLU mmol/L	BIL μmol/L	KET mol/L	SG	BLO Ery/μL	pH	PRO g/L	URO μmol/L	NIT	LEU leu/μL	Color	Clarity
0	–	–	–	1.01	–	8.5	–	3.2	–	–	Yellow	Clear
1	–	–	–	1.02	Trace	7.0	–	3.2	–	–	Yellow	Clear
2	–	–	–	1.02	Trace	8.5	–	3.2	–	–	Yellow	Clear
3	–	–	–	1.02	Trace	8.5	–	3.2	–	–	Yellow	Clear
4	–	–	–	1.01	–	≥ 9	Trace	3.2	–	–	Yellow	Clear
5	–	–	–	1.01	–	≥ 9	–	3.2	–	–	Yellow	Clear
6	–	–	–	1.01	Trace	≥ 9	1	3.2	–	–	Yellow	Clear
7	–	–	–	1.02	Trace	≥ 9	–	3.2	–	–	Yellow	Clear
8	–	–	–	1.01	–	≥ 9	–	3.2	–	–	Yellow	Clear
9	–	–	–	1.02	–	8.5	–	3.2	–	–	Yellow	Clear
10	–	–	–	1.01	–	≥ 9	0.3	3.2	–	–	Yellow	Clear
11	–	–	–	1.01	Trace	7.5	–	3.2	–	–	Yellow	Clear
12	–	–	–	1.02	–	8.5	1	3.2	–	–	Yellow	Clear

*GLU* Glucose, *BIL* Bilirubin, *KET* Ketone, *SG* Specific Gravity, *BLO* Occult Blood, *PRO* Protein, *URO* Urobilinogen, *NIT* Nitrite, *LEO* Leukocyte

**Table 4:** Urine test of the PEG-GNRs treated *Macaca fascicularis* (number 4) in 12 weeks

Date Week	GLU mmol/L	BIL μmol/L	KET mol/L	SG	BLO Ery/μL	pH	PRO g/L	URO μmol/L	NIT	LEU leu/μL	Color	Clarity
0	–	–	Trace	1.01	–	≥ 9	–	3.2	+	–	Yellow	Clear
1	–	–	–	1.02	–	8.5	–	3.2	–	–	Yellow	Clear
2	–	–	–	1.01	Trace	≥ 9	1	3.2	–	–	Yellow	Clear
3	–	–	Trace	1.01	–	≥ 9	–	3.2	+	–	Yellow	Clear
4	–	–	–	1.01	Trace	≥ 9	1	3.2	–	–	Yellow	Clear
5	–	–	–	1.02	–	≥ 9	–	3.2	–	–	Yellow	Clear
6	–	–	–	1.02	Trace	≥ 9	Trace	3.2	Trace	–	Yellow	Clear
7	–	–	Trace	1.01	Trace	≥ 9	0.3	3.2	–	–	Yellow	Clear
8	–	–	–	1.01	–	7.5	0.3	3.2	–	–	Yellow	Clear
9	–	–	–	1.01	–	≥ 9	–	3.2	–	–	Yellow	Clear
10	–	–	–	1.02	5	8.5	Trace	3.2	–	–	Yellow	Clear
11	–	–	–	1.01	Trace	8.5	–	3.2	–	–	Yellow	Clear
12	–	–	–	1.01	–	8.5	–	3.2	–	–	Yellow	Clear

*GLU* Glucose, *BIL* Bilirubin, *KET* Ketone, *SG* Specific Gravity, *BLO* Occult Blood, *PRO* Protein, *URO* Urobilinogen, *NIT* Nitrite, *LEO* Leukocyte

## Materials and methods

### Materials

Chloroauric acid (HAuCl_4_·3H_2_O) and cetyltrimethylammonium bromide (CTAB) were purchased from Sinopharm Chemical Reagent (Shanghai, China) and China National Medicines (Beijing, China), respectively. Thiolated-PEG (mPEG_5000_-SH) was purchased from Sigma-Aldrich (USA). L-ascorbic acid (Vc) and silver nitrate (AgNO_3_) were purchased from Alfa Chemicals (UK). Sulfuric acid (H_2_SO_4_), nitric acid (HNO_3_), hydrochloric acid (HCl), and perchloric acid (HClO_4_) were obtained from KeLong Chemicals Reagent (Chengdu, China). All reagents were of analytical grade.

### The preparation and characterization of CTAB-GNRs and PEG-GNRs

We prepared CTAB-GNRs using a modified version of our previously published seed-mediated approach [54]. The concentration of Au in CTAB-GNRs dispersion was determined via lysis (HNO_3_: HCl: HClO_4_ = 3:1:2) and ICP-AES analysis (SPECTRO ARCOS, Spectro, Germany).

The surface molecule CTAB was then replaced with mPEG_5000_-SH. The CTAB-GNRs were purified via centrifugation twice and redispersed in deionized water to a final concentration of [Au] of 1 mg/mL. Then, 200 mg thiolated-PEG in 32 mL water was added to the CTAB-GNRs and the mixture was gently stirred overnight. The molar ratio of PEG-SH and Au was 0.20. Finally, the obtained PEG-GNRs were purified by centrifugation twice.

The CTAB-GNRs and PEG-GNRs were characterized by TEM, AFM, UV–Vis, and *zeta* potentials as described elsewhere [4, 54].

### Small animal study

The study was approved by the ethics committee of the State Key Laboratory of Oral Diseases and conducted in compliance with the animal care and use guidelines of Sichuan University. Male SD rats (6–8 weeks old) were purchased from the Experimental Animals Center of Sichuan Province, China. The rats were categorized into four groups. The control group was injected with normal saline, and the other groups were administered 5, 10, and 20 mg/kg of PEG-GNRs by tail vein injection. At 0.25, 0.75, 1, 2, 6, 12, 24, and 48 h, tail vein blood samples were obtained to determine the concentrations of Au in the blood. Blood markers were assessed at 3 days and at 1 week and 2 weeks post-injection. The major organs were lysed, and PEG-GNRs was detected by ICP-AES. After 2 weeks, all rats were sacrificed and the major organs were collected for H&E staining.

### *Macaca fascicularis * study

The *M. fascicularis* study was supported by West China-Frontier Pharma Tech Co. (WCFP), National Chengdu Center for Safety Evaluation of Drugs (NCCSED), China. The protocol was reviewed and approved by the ethics committee of NCCSED. Four adult male *M. fascicularis* (age 3–4 years, body mass was ~ 4.0 kg) were used in the toxicity study (3 were treated with PEG-GNRs and 1 was considered the control). Animals were individually housed in stainless steel cages and fed a commercial monkey diet from local supplies. Water was available ad libitum. The professional research staff regularly inspected the animals twice a day.

The animals received 1.0 mg/kg of PEG-GNRs by intravenous drip over 30 min. The control animal was administered normal saline. Peripheral blood samples were collected at designated times to determine the blood clearance of PEG-GNRs and other parameters. The blood markers analysis was conducted on a Siemens Advia 2120 and a Roche Cobas 6000-C501. Every week, 3–5 mL of urine was collected for urinalysis on a Siemens Clinitek Status machine. Body mass, body temperature, blood pressure, appearance, and exploratory behavior of the Macaca fascicularis were simultaneously recorded. After 12 weeks, the animals were sacrificed for tissue analysis. The heart, liver, spleen, lungs, kidneys, brain, lymph, intestine, skeleton muscle, and skin were collected. The organs were lysed to measure the amount of Au by ICP-AES. Meanwhile, the remaining tissues were fixed for obtaining H&E stained histological sections and examined with an Olympus BX60 microscope.

## Conclusions

This study broadened the study of systemic PEG-GNRs toxicity research from rodents to non-human primates in a preclinical test. We detected a negligible immune response in the blood marker analysis of *M. fascicularis*. This immune response quickly disappeared suggesting a normal immune reaction to any foreign body. All the animals appeared healthy during the 12-week study period, supported by normal blood, urine, body temperature, body masses, and blood pressure levels. Biodistribution analysis showed that PEG-GNRs primarily accumulated in the RES system, but histological analysis revealed no apparent damage to these organs. Overall, the PEG-GNRs showed excellent biocompatibility in both rats and *M. fascicularis* at clinically relevant doses. The non-human primate safety evaluation of PEG-GNRs showed great potential for human clinical application. More detailed, long-term investigations of GNRs in higher mammals are required.

## Supplementary Information

**Additional file 1: Figure S1.** The bio-distribution at 3 days, 1 week, and 2 weeks after systemic administration of (**a**) 5 mg/kg and (**b**) 20 mg/kg PEG-GNRs in rats.** Figure S2** H&E staining photographs of major organs in rats at 2 weeks after treatment. Images were acquired at 200× magnification. **Figure S3.** Blood markers test for Macaca fascicularis treated with PEG-GNRs.** Figure S4.** Histological images of major organs of the control and Macaca fascicularis (number 1) post-injection at 12 weeks. Images were acquired at 200× magnification.** Figure S5.** Histological images of the major organs of Macaca fascicularis number 2 and number 3 post-injection at 12 weeks.

## Data Availability

All data are available in the main manuscript and supplementary information.
