# Supplementary material for: Broadening the biocompatibility of gold nanorods from rat to Macaca fascicularis: advancing clinical potential
Source: J Nanobiotechnology. 2021 Jun 30;19:195. doi: 10.1186/s12951-021-00941-1 (PMC8243831; doi:10.1186/s12951-021-00941-1)
Supplement: Supplementary file 1 — Additional file 1: Figure S1. The bio-distribution at 3 days, 1 week, and 2 weeks after systemic administration of (a) 5 mg/kg and (b) 20 mg/kg PEG-GNRs in rats. Figure S2 H&E staining photographs of major organs in rats at 2 weeks after treatment. Images were acquired at 200× magnification. Figure S3. Blood markers test for Macaca fascicularis treated with PEG-GNRs. Figure S4. Histological images of major organs of the control and Macaca fascicularis (number 1) post-injection at 12 weeks. Images were acquired at 200× magnification. Figure S5. Histological images of the major organs of Macaca fascicularis number 2 and number 3 post-injection at 12 weeks. [file 12951_2021_941_MOESM1_ESM.docx]

**Supporting Information**

**Broadening the Biocompatibility of Gold Nanorods from Rat to *Macaca fascicularis*: Advancing Clinical Potential**

Jinfeng Liao^1, #^, Taorang Tian^1, #^, Sirong Shi^1^, Xueping Xie^1^, Shuanglin Peng^2^, Ying Zhu ^3, 4,^ *, Jingang Xiao^2^*, Yunfeng Lin^1, 5^*

^1^ State Key Laboratory of Oral Diseases, National Clinical Research Centre for Oral Diseases, West China Hospital of Stomatology, Sichuan University, Chengdu, 610041, China.

^2^ Department of Oral and Maxillofacial Surgery, The Affiliated Stomatology Hospital of Southwest Medical University, Luzhou 646000, China.

^3^ Division of Physical Biology, CAS Key Laboratory of Interfacial Physics and Technology, Shanghai Synchrotron Radiation Facility, Shanghai Institute of Applied Physics, Chinese Academy of Sciences, Shanghai 201800, China

^4^ Zhangjiang Laboratory, Shanghai Advanced Research Institute, Chinese Academy of Sciences, Shanghai 201210, China

^5^College of Biomedical Engineering, Sichuan University, Chengdu, 610041, China

*Corresponding author.

1. mail: [yunfenglin@scu.edu.cn](mailto:yunfenglin@scu.edu.cn) (Y. Lin); drxiaojingang@163.com (J. Xiao); zhuying@zjlab.org.cn (Y. Zhu)

^#^These authors contributed equally to this work.


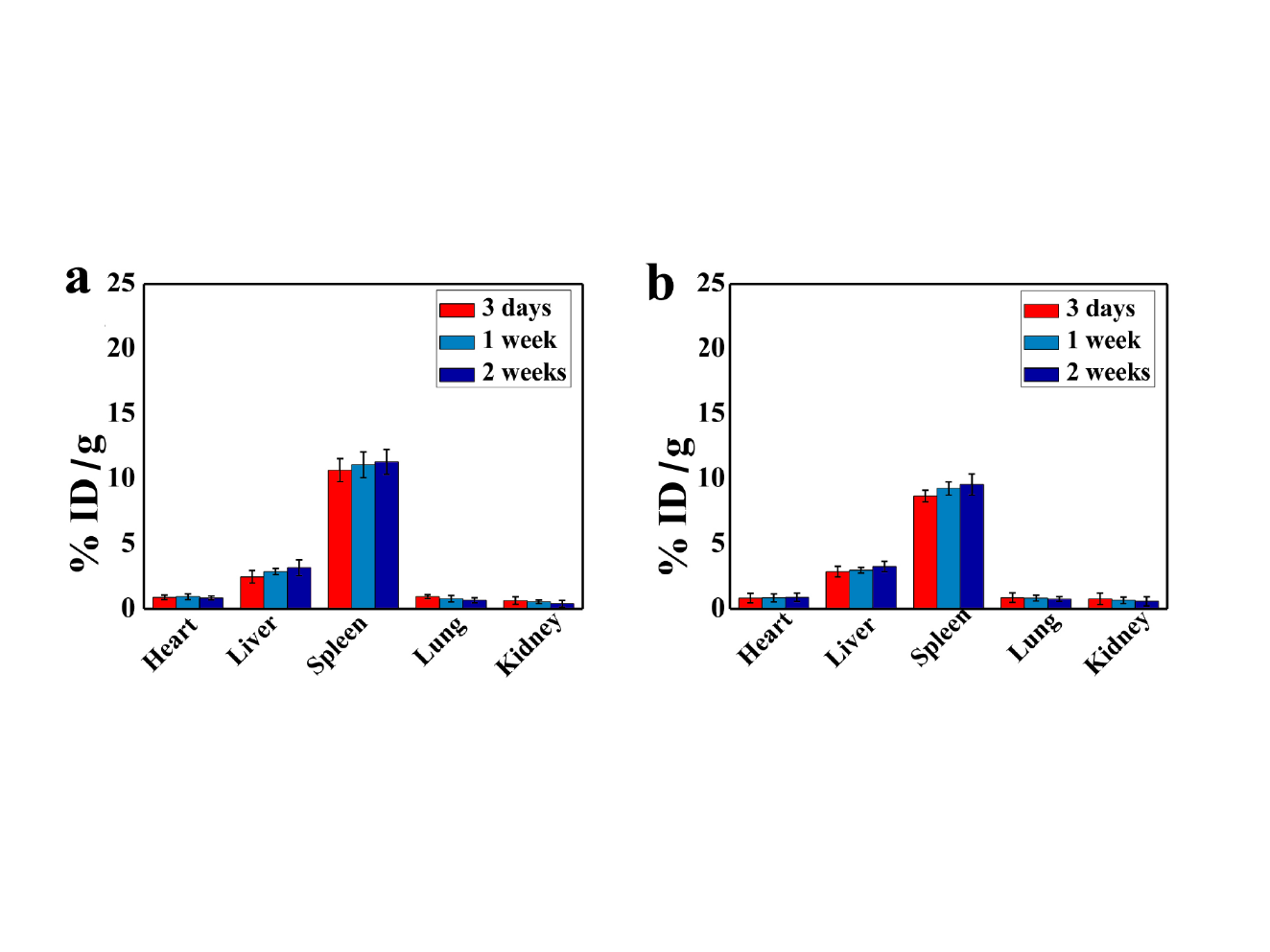


**Figure S1.** The bio-distribution at 3 days, 1 week, and 2 weeks after systemic administration of (a) 5 mg/kg and (b) 20 mg/kg PEG-GNRs in rats.


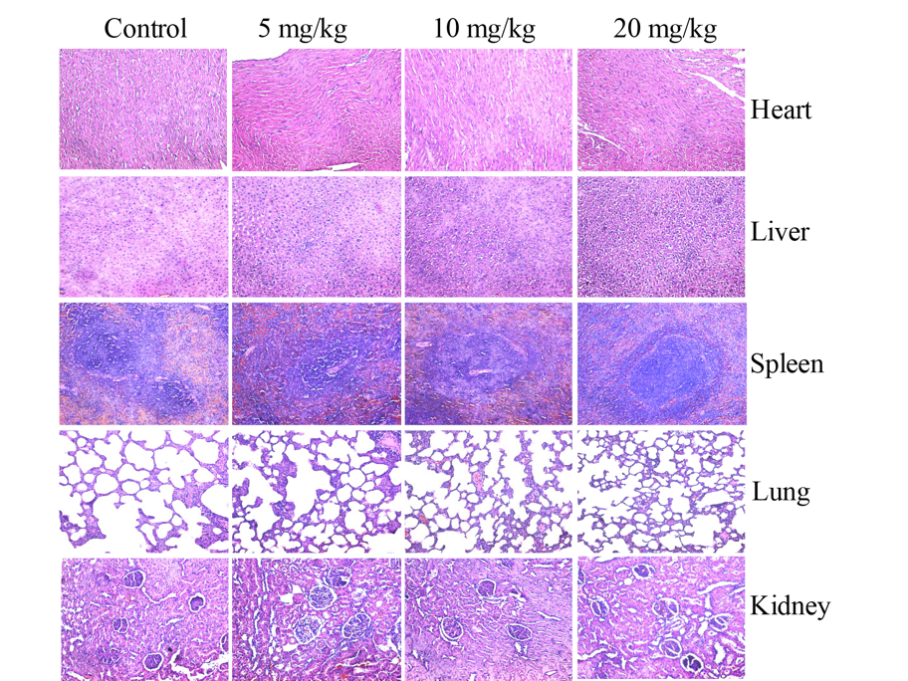


**Figure S2.** H&E staining photographs of major organs in rats at 2 weeks after treatment. Images were acquired at 200× magnification.


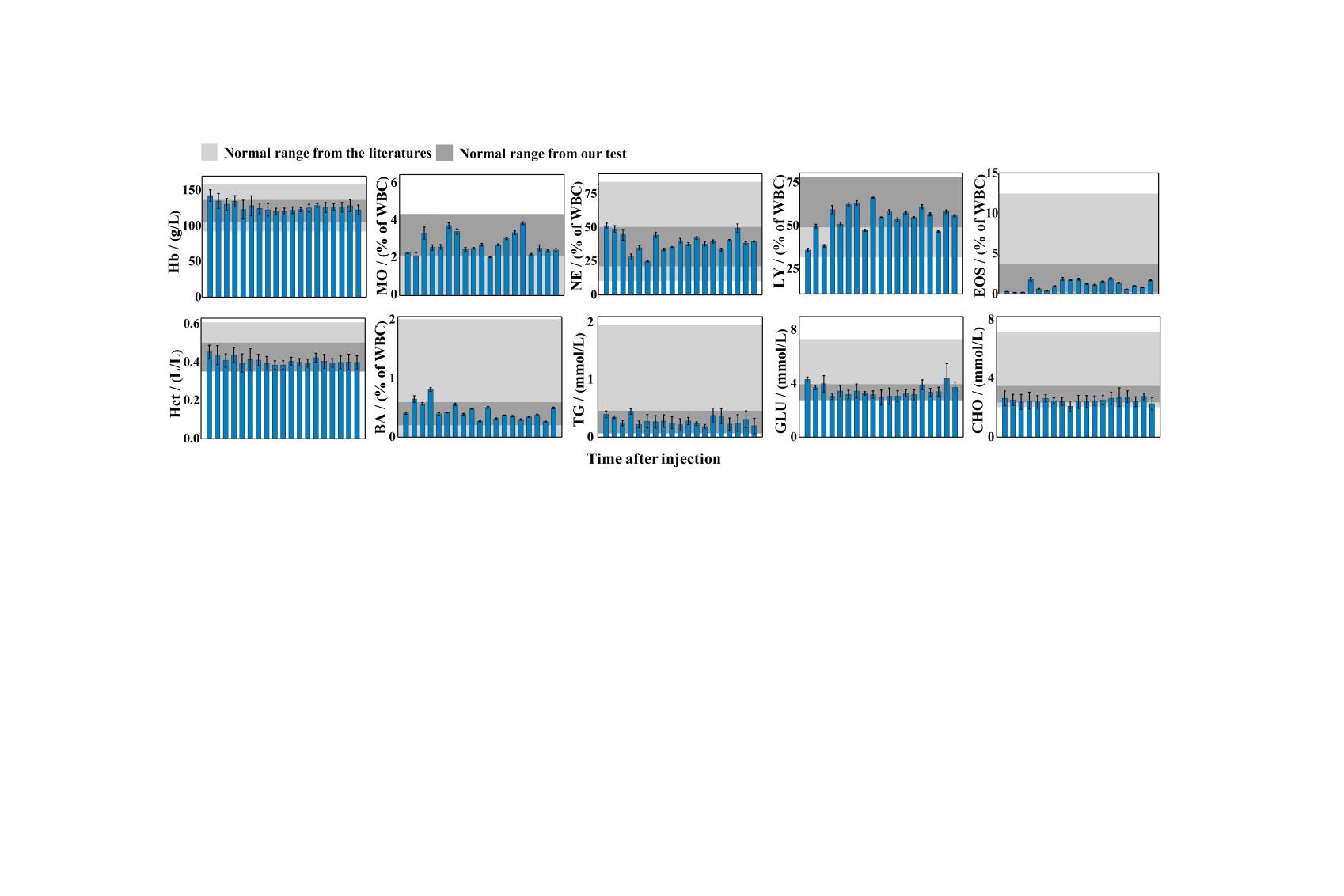


**Figure S3.** Blood markers test for *Macaca fascicularis* treated with PEG-GNRs. Times after injection in the figures from left to right are 1 h, 2 h, 6 h, 12 h, 1 d, 2 d, 3 d, 1 w, 2 w, 3 w, 4 w, 5 w, 6 w, 7 w, 8 w, 9 w, 10 w, 11 w, and 12 w. Abbreviation: hemoglobin, Hb; monocyte, MO; neutrophil granulocyte, NE; lymphocyte, LY; eosinophil granulocyte, EOS; hematocrit, Hct; basophil granulocyte, BA; triglyceride, TG; blood glucose, GLU; total cholesterol, CHO.


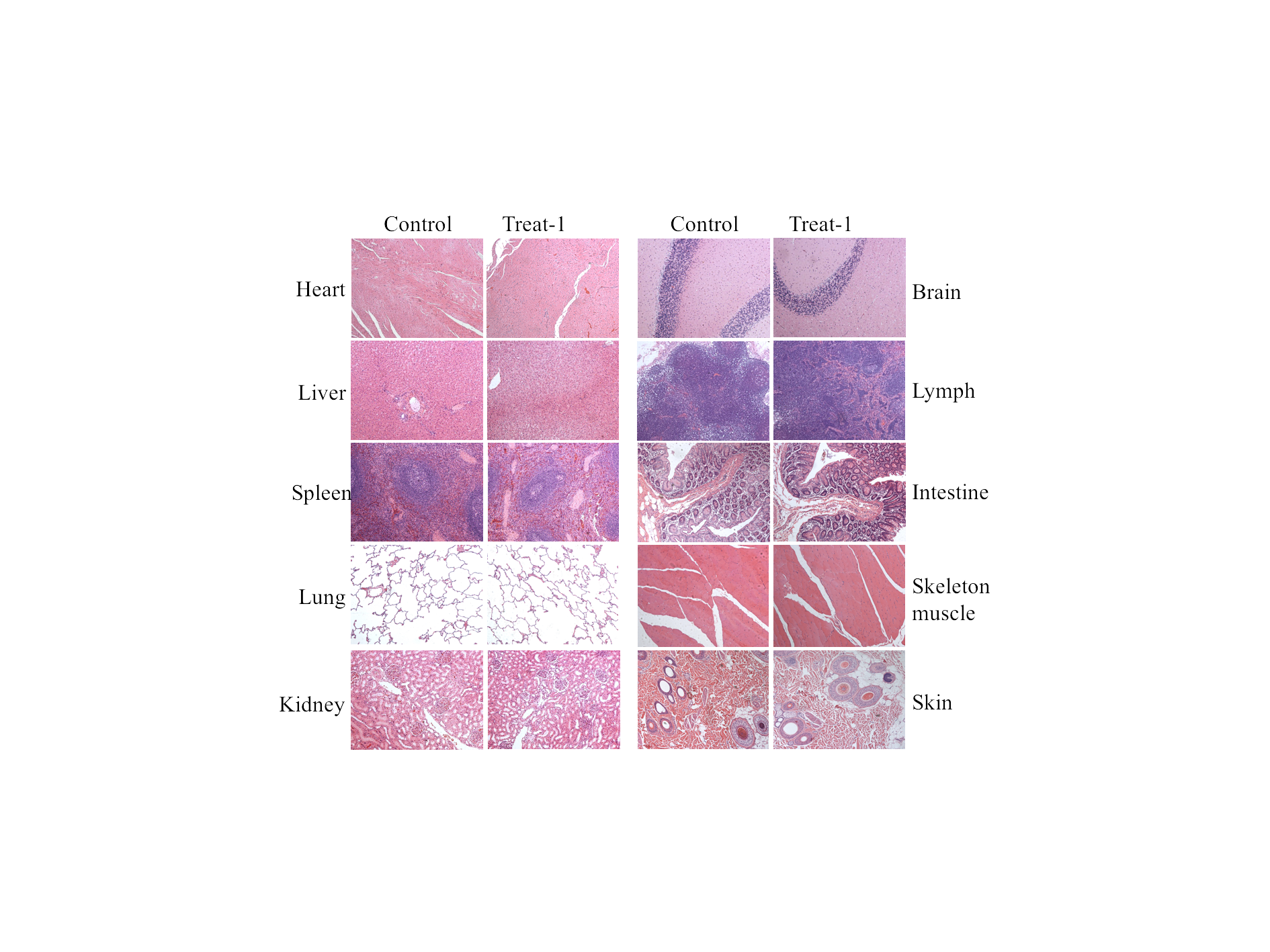


**Figure S4.** Histological images of major organs of the control and *Macaca fascicularis* (number 1) post-injection at 12 weeks. Images were acquired at 200× magnification.


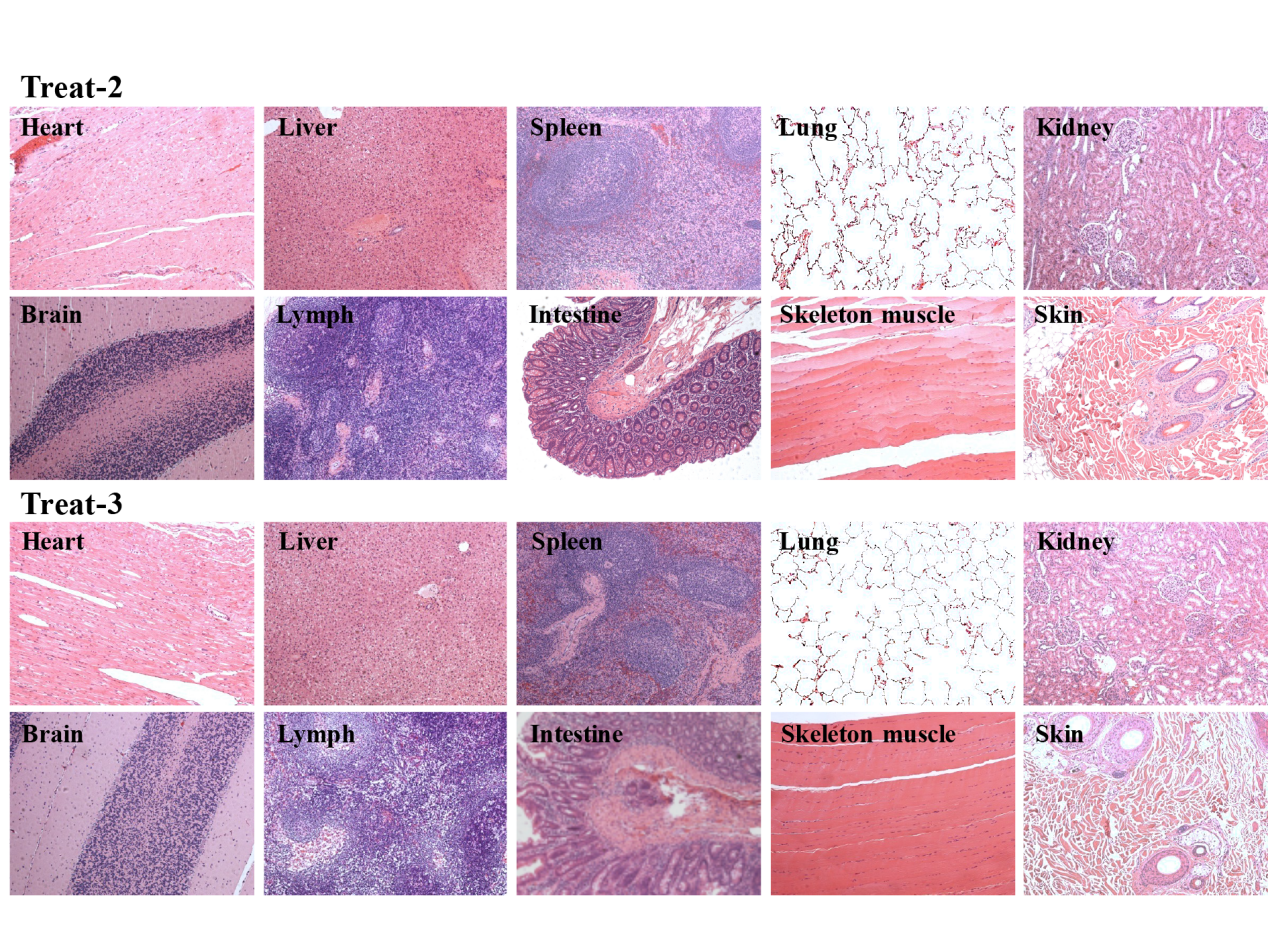


**Figure S5.** Histological images of the major organs of *Macaca fascicularis* number 2 and number 3 post-injection at 12 weeks. No abnormal was observed in the tissues. The images were taken at 200× magnification.
